# Time-resolved and parameter-free measures of spike train synchrony: properties and applications

**DOI:** 10.1186/1471-2202-16-S1-P133

**Published:** 2015-12-18

**Authors:** Mario Mulansky, Nebojsa Bozanic, Thomas Kreuz

**Affiliations:** 1 Institute for Complex Systems, CNR, Sesto Fiorentino, 50019, Italy

## 

The relevance of exact spike timings in neural coding was presumed since a long time and has now been experimentally established, see e.g. in [1,2]. A popular approach to the analysis of spike timings is to measure the synchrony of spike trains. With the recent advancements of the experimental techniques, it is now possible to simultaneously record the activity of hundreds of neurons. The analysis of such collective responses requires new mathematical tools that are able to detect synchrony in groups of spike trains. Here, we present three methods to quantify spike train synchrony that are applicable in such multivariate situations. All of these methods are parameter-free and time-resolved which makes them easy to handle and able to detect temporal changes of synchrony.

**Figure 1:**
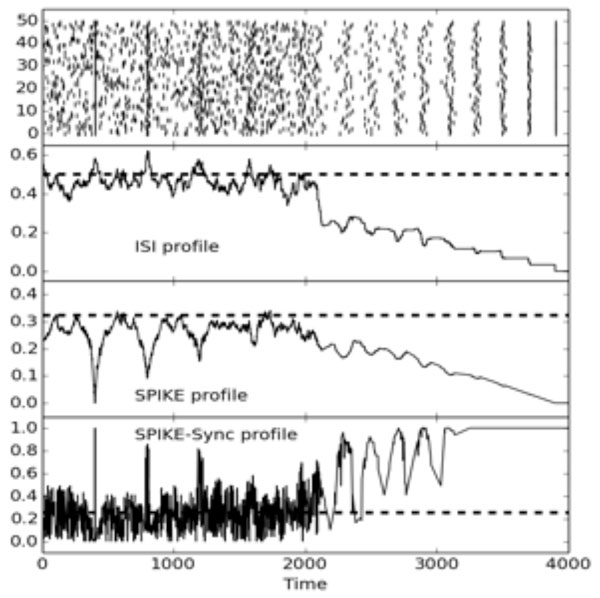
**Multivariate ISI, SPIKE and SPIKE-Synchronization profiles for 50 artificially generated spike trains**. The dashed lines represent the respective expectation values for random Poisson spike trains.

Specifically, we discuss the ISI-distance [3], the SPIKE-distance [4] and the very recently proposed SPIKE-Synchronization [5]. The ISI-distance is based on the relative differences of interspike intervals, while the SPIKE-distance uses exact spike timings. SPIKE-Synchronization can be understood as a time-resolved, spike-wise coincidence detector. Figure 1 shows exemplarily the time-resolved profiles of all three methods for 50 artificially created spike trains.

We analyze the mathematical properties of all three measures and discuss their advantages and disadvantages [6]. Specific focus lies on the statistical relevance of the obtained values compared to random spike trains. By calculating the expectation values for Poisson spike trains we are able to provide an important point of reference for interpreting numerical and experimental results. Finally, we show exemplary applications of the methods to spike trains obtained from numerical simulations [7] as well as experimental recordings [4].

The methods are implemented in both the Matlab-based graphical user interface SPIKY [5] and the Python library PySpike.
